# Changes in the Use of Emergency Care for the Youth With Mental Health Problems Over Decades: A Repeated Cross Sectional Study

**DOI:** 10.3389/fpsyt.2019.00026

**Published:** 2019-02-06

**Authors:** Xavier Benarous, Vanessa Milhiet, Alice Oppetit, Sylvie Viaux, Nadjia Mahi El Kamel, Vincent Guinchat, Jean-Marc Guilé, David Cohen

**Affiliations:** ^1^Department of Child and Adolescent Psychiatry, Pitié-Salpêtrière Hospital, Paris, France; ^2^INSERM Unit U1105 Research Group for Analysis of the Multimodal Cerebral Function, University of Picardy Jules Verne (UPJV), Amiens, France; ^3^Department of Psychiatry, McGill University, Montreal, QC, Canada; ^4^Child and Adolescent Psychopathology Department, Amiens University Hospital, Amiens, France; ^5^CNRS UMR 7222, Institute for Intelligent Systems and Robotics, Sorbonnes Université, Paris, France

**Keywords:** emergency unit, prevalence, mental health, adolescents, mental health services, crisis intervention, suicidal behaviors

## Abstract

To understand whether changes exist in the types of youths mental health problems addressed in emergency in a context of increasing demand, we conducted a retrospective chart review in an emergency care outpatient unit. Data from children and adolescents admitted at four different time periods (years 1981, 1992, 2002, and 2017) were compared to determine trends in terms of patients' characteristics, nature of the mental health problems and final care decisions. Between 1981 and 2017 there was a 3.85 times increase in the annual number of patients presenting to the emergency consultations. The proportion of youths being referred for anxiety or depressive symptoms sharply increased over time, while no differences were found for the proportion of aggressive behaviors and suicidal attempts. Anxiety disorders became the most frequent discharge psychiatric disorder in youths admitted in the emergency unit, rising from 5% in 1981 to 34% in 2017. Significant changes were also observed in the source of referral to the emergency unit; in particular emergency consultations in 2017 were about twice as likely as in 1981 to be requested directly by the family. This data suggested that the increased use of emergency services observed over the last decades is associated with significant changes in the patient and his/her family's demands about mental health difficulties. Such findings are worth considering for mental health interventions that aim to address the emergency overcrowding issue.

## Introduction

A clear increase of the use of emergency services by children and adolescents with mental health problems has been observed in most developed countries over the last decades (1–6). For example, in the US the number of visits to Emergency Departments (EDs) for mental health difficulties in children nearly doubled during the past 15 years (3). Actually, EDs became the primary point of entry to the mental health care system for a majority of help-seeking youth (7, 8). This represents a significant challenge for the emergency structures that have to meet steadily growing demands with stable resources (1–3, 7).

An increase in the prevalence of pediatric psychiatric disorders is unlikely to alone provide such a significant rise in the number of emergency consultations. The shortage of mental health providers since 1980s and the resulting saturation of the outpatient care service were also brought up as a possible explanation (7). For example, in France the total number of practicing child and adolescent psychiatrists was decreased by half over the last decade. During the same period, the number of children followed in outpatient structures has doubled, with a subsequent increase of the average wait time for an appointment for a child with a psychiatrist (9). A lack of child and adolescent psychiatrist to meet the population need have been observed in US (1, 3), Canada (10), and other European (11) countries.

Another research perspective to explain this global trend is to examine how the type of the youth mental health problems addressed in EDs has changed over time (4, 5, 12–16). A better knowledge of the reasons and source of the referral to the emergency structure is of main interest to understand the patient and the family needs. It may help to develop more-targeted interventions in order to address some of these difficulties and ultimately limit the emergency-unit overcrowding problem (7). However, so far the evolution of the demographic and clinical features of children and adolescents using emergency resources for mental health problems has received little empirical scrutiny (4, 5, 12, 13, 15, 16).

The specificities of each national pediatric mental health system make it difficult to generalize findings from studies conducted in US samples (4, 5, 12, 15, 16) in a European context. In particular, in most European countries access to outpatient care is completely free of charge for the patient and his/her family. In other words, there is no financial reason for a family to consult in ED rather than in outpatient facilities. In this way, a study conducted in a European sample provides a natural opportunity to control the impact of financial reasons to understand the rise in emergency consultations by children and adolescents. Besides, the time periods of previous published studies conducted in EDs were limited to few years, exceptionally one decade (4, 5). A longer time perspective may help to better understand how the use of emergency care for youth with mental health problems has evolved in a context of mental health system reorganization since 1970s (9, 17).

In this paper we investigated the changes in the types of youth mental health problems observed in an emergency unit of a child and adolescent psychiatric department. Temporal trends were studied over more than three decades using multiple cross-sectional data across four time points (1981, 1992, 2002, and 2017).

The first aim of this study was to determine the evolution of the annual number of emergency consultations in a tertiary psychiatry care unit from 1981 to 2017. We hypothesized an increase in the number of consultation demands over the study period. The rise was expected to be around 2- to 5- fold and to be consistent with prior reports (4–6, 12–15). If this is true, it will support the assumption that an economic reason is not a determining factor of the progression of the use of emergency services by children and adolescents.

The second aim of this study was to examine the changes in the sample characteristics, the source and the main reason for the consultation, the main discharge psychiatric diagnosis and the final care decision over the study period. Two hypotheses were specifically tested.

- Firstly, it has been thought that the saturation of community mental health resources contributes to the rise of emergency consultations (1, 7, 9, 15), i.e., youths with non-urgent clinical problems might go to emergency structures to avoid delay in consultations. Here we expected that the use of emergency consultations for common/more frequently benign cause (e.g., anxiety) increased to a higher extent relative to more frequently severe causes (e.g., delusion). This would be in line with prior observations based on discharge diagnosis (15) and dedicated measure of clinical severity (4).
- Secondly, previous longitudinal studies showed a decrease over the last decades of the rate of admission in inpatient units for children and adolescents following an emergency visit (3, 4, 10). A similar result was expected in our sample with a decreased proportion of youths directly admitted to an inpatient service following the visit from 1981 to 2017.

For other variables no apriori hypotheses were set up and an exploratory approach was used.

## Materials and Methods

The Child and Adolescent Psychiatric Department of the Pitié-Salpêtrière Hospital (CAPD-PSH) is a tertiary care referral center providing care to children and youths up to 18 years of age, with the exception of certain young adults with ongoing pediatric subspecialty needs. It is the largest CAPD in the Paris area. It includes six inpatient units (67 beds), 3 day care hospitals (40 patients), and also provides many outpatient consultations (≥20,000/year). The service catchment area is around 280,000 people under 18 years old. The emergency consultation service at the CAPD-PSH has existed since 1972. The service is open on weekdays during daily hours and Saturday morning. It is located within the main building of the CAPD-PSH. Each new patient has a primary assessment by one member of the paramedical team prior to the psychiatric assessment conducted by a senior psychiatrist. No triage or acuity assessments are made on site, however a first screening can occasionally be conducted by phone by a specialized nurse to redirect non-urgent situations (e.g., information request). The average length of duration for the visit is around 1–3 h and no bed is specifically dedicated for an extended period of observation. After the visit, the clinician in charge of the patient enters clinical information in a specific database.

Data from the electronic medical record of all patients admitted in the emergency unit at CAPD-PSH between 1st of December 2016 and 1st of December 2017 were extracted. Data were compared with information previously obtained from subjects referred to the emergency unit in 1981 (18), in 1992 and in 2002 (19). Data were systematically collected by the psychiatrist involved using a standardized questionnaire to document demographic features (age, gender), nature of the referral (source and main reason for referral), the discharge psychiatric diagnosis, and the final care decision. The questionnaire has been computerized since 2012. The psychiatric diagnosis was coded by using ICD-10-CM and then grouped into 11 categories based on a modification of the Diagnostic and Statistical Manual of Mental Disorders, 4th edition classification (20). To facilitate the comparison with data previously extracted, we selected for each case in 2017 the main reason for referral. When subjects received two reasons for referral, we considered only the one which had the greater influence on the clinical decision. For example, if a child presented both “suicidal thought” and “school failure,” the first was considered as the main reason for referral. To keep in line with the method used by Blondon et al. (19), when two or more visits were recorded for a patient only admissions that occurred at least 3 months after the initial visit were taken into account.

The process of care for emergency consultation remained roughly consistent over the study period [described in (18, 19)]. The category “post-emergency consultation” refers to a second consultation in the emergency unit within 72 h usually with the same clinician. The category “delay hospitalization” refers to the patient orientation to a pediatric unit in a general hospital until the admission to a specialized unit for child and adolescent psychiatry. The data extracted have been anonymized. The study was reviewed and determined to be exempt from full review by the institutional review board at our institution. The study is in line with the Helsinki convention and the ethical general recommendation regarding chart review (21).

No exclusion criteria were used. Of note, the following groups were not included in this study. First, patients above 15 who need psychiatric assessment after 7 p.m. and/or the weekend were admitted in the adult ED of the Pitié-Salpêtrière Hospital. However, during daily hours on the week and Saturday morning patients above 15 who need psychiatric assessment were addressed to the emergency unit at the CAPD-PSH. This repartition between the CAPD and adult ED at the PSH did not change during the study period. Second, patients transferred from another hospital directly to an inpatient unit of the CAPD-PSH were not assessed to the emergency consultation unit.

We used descriptive statistics to summarize our results. Continuous variables such as age are presented with means ± standard deviation. Proportions such as rate of admission are presented with percentages. Data were statistically analyzed using R software. Bivariate analyses were conducted using χ^2^ for categorical variables, and test-t for continuous variables. Chi-2 tests were used to compare proportions in the four independent samples (1981, 1992, 2002, and 2017). Exploratory analyses were conducted to compare the demographic and clinical profiles of children and adolescents using a cut-off age of 13.

## Results

### Demographics Characteristics

The annual number of emergency consultations continuously increased over time (Table 1). From 1981 to 2017 there was a 3.85 time increase in patient referrals, with a 131% increase between 1981 and 1992, a 9% increase between 1992 and 2002, and a 37% increase between 2002 and 2017. The differences between the mean ages of subjects over time were statistically significant (χ^2^ = 21.867, *df* = 2, *p* < 0.001). On average, youths admitted in 1981 were 1 year 7 months older than those admitted in 2017. Gender ratio was stable over time, around 0.55 for male.

**Table 1:** Demographic characteristics of patients admitted to the emergency consultation service at the CAPD-PSH in 1981, 1992, 2002, and 2017.

	**Year**				
	**1981**	**1992**	**2002**	**2017**	***p***
Annual number of emergency consultations	85	196	214	294	< 0.001
Mean age ±*SD* [min – max]	15.4	13.7 ± 3.9 [2 – 20]	12.3 ± 4.1 [2 – 18]	13.8 ± 3.1 [2 – 19]	< 0.001
Gender, male, *n* (%)	NA	104 (53%)	116 (54%)	161 (55%)	0.933

### Source and Main Reason for Referral

The comparison of the source of referral for an emergency visit over time is presented in Table 2. The proportion of consultations requested by the family and/or relatives almost doubled between 1981 and 2017 (respectively 29.4 and 57.8% of the annual number of visits). The consultations at the youth's request represented 6% of the total number of referrals in 1981 but only 0.3% in 2017.

**Table 2:** Source and main reason for referral to the emergency consultation service at the CAPD-PSH in 1981, 1992, 2002, and 2017.

	**Year**				
	**1981 (*N* = 85)**	**1992 (*N* = 196)**	**2002 (*N* = 214)**	**2017 (*N* = 294)**	***p***
**SOURCE OF REFERRAL**					
At the youth's request	6.0%	2.0%	2.5%	0.3%	0.07
Family and/or relatives	29.4%	23.5%	55.4%	57.8%	< 0.001
Medical professional	24.7%	26.5%	15.6%	14.3%	0.002
Psychiatrist or psychologist	8.2%	21.9%	12.4%	10.5%	0.001
Emergency department	4.7%	6.1%	1.9%	2.0%	0.044
Police	12.9%	1.0%	0.6%	1.0%	< 0.001
School professionals	9.4%	16.8%	10.5%	12.4%	0.189
Other	4.7%	2.0%	1.0%	1.7%	0.204
**MAIN REASON FOR REFERRAL**					
Suicidal attempt and/or NSSI	–	12.7%	11.8%	9.2%	0.428
Anxiety	–	5.1%	10.8%	21.1%	< 0.001
Depressive mood	–	5.1%	15.9%	16.7%	< 0.001
Delusion and/or dissociation	–	8.7%	6.1%	4.4%	0.15
Physical complaints and/or psychosomatic problems	–	13.3%	5.7%	5.1%	0.002
Substance abuse	–	1.5%	3.2%	3.7%	0.355
Agitation and/or aggressive behavior	–	31.1%	28.3%	23.8%	0.187
Runaway and/or wandering	–	6.1%	1.9%	3.4%	0.074
School problems and/or learning difficulties	–	6.1%	2.2%	1.7%	0.015
Physical and/or sexual abuse	–	1%	4.1%	1.0%	0.038
Familial crisis	–	2%	4.8%	6.1%	0.101
Relational difficulties	–	2.6%	2.2%	1.4%	0.585
Other	–	4.6%	2.9%	2.4%	0.381

*Data for the main reason for referral were not systematically collected in 1981; NSSI, Non-suicidal self injury*.

**Figure 1:**
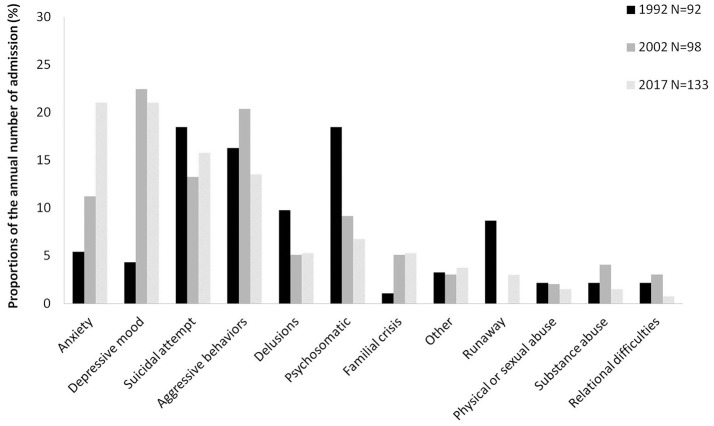
Main reason for referral to the emergency consultation service at the CAPD-PSH among girls in 1992, 2002, and 2017.

**Figure 2:**
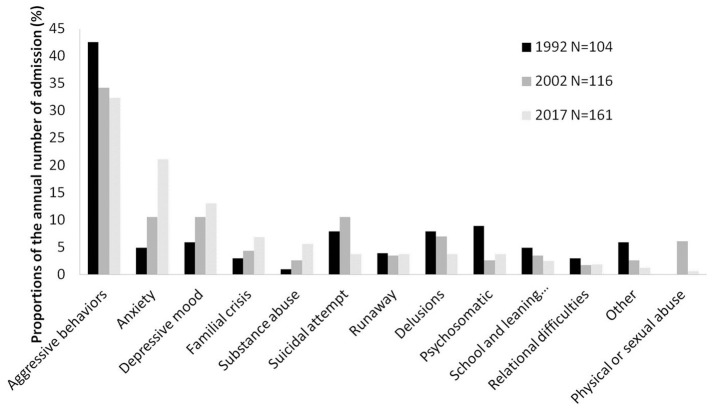
Main reason for referral to the emergency consultation service at the CAPD-PSH among boys in 1992, 2002, and 2017.

Agitation and/or aggressive behavior were the main reason for referral to the emergency consultation service for all the periods considered (around a quarter of the visits). The consultations for suicidal attempt and/or non-suicidal self-injury (NSSI) decreased from 12.7% in 1992 to 9.2% in 2017. The referral for anxiety and depressive mood sharply increased over time with, respectively a four times and a three times increase between 1992 and 2017. The referral for school problems and/or learning difficulties decreased from 6.1 to 1.7% during the same period. The main reason for referral differed across gender (Figures 1, 2).

### Main Psychiatric Diagnosis at Discharge

The annual number of youths diagnosed with an anxiety disorder as the primary psychiatric disorder sharply increased over time (Table 3). This diagnosis was seven times more likely in 2017 compared to 1992. A substance use disorder was four times more frequent in the youths admitted in the emergency consultation service in 2017 compared to 1992. The proportion of youths with a discharge diagnosis of schizophrenia or other psychotic disorder in 2017 was half of that in 2002.

**Table 3:** Discharge psychiatric diagnosis of youths admitted to the emergency consultation service at the CAPD-PSH in 1992, 2002, and 2017.

	**Year**			
	**1992 (*N* = 196)**	**2002 (*N* = 214)**	**2017 (*N* = 294)**	***p***
**PSYCHIATRIC DIAGNOSES**				
Anxiety disorder	5.2%	19.3%	34.0%	<0.001
Depressive disorder	24.2%	35.8%	23.5%	0.004
Manic or mixed episode	1.0%	1.9%	0.7%	0.448
Disruptive behavior disorder	21.1%	20.8%	19.7%	0.919
Substance use disorder	1.0%	2.4%	4.1%	0.111
Schizophrenia or other psychotic disorder	6.2%	8.0%	3.1%	0.048
Pervasive developmental disorder	6.2%	1.9%	5.8%	0.064
Intellectual disability	2.6%	1.4%	2.0%	0.698
Eating disorder	6.2%	1.9%	1.0%	0.002
Personality disorder	9.8%	1.9%	4.1%	0.001
No psychiatric disorder	5.2%	3.3%	1.4%	0.054
Other	11.3%	1.4%	0.7%	<0.001

*The discharge psychiatric diagnoses were not systematically collected in 1981*.

### Final Care Decision

The admission to an inpatient unit directly following the emergency visit represented three-quarters of the total annual number of consultation in 1981 but only 15.6% in 2017 (Table 4). In absolute terms, this decrease represents 64 annual admissions in 1981 vs. 48 in 2017. In parallel, the orientation toward outpatient services became the most frequent outcome. In 2017 59% of the annual visits were directed to a community mental health service and 13.3% were addressed to a post-emergency consultation.

**Table 4:** Orientation after the visit to the emergency consultation service at the CAPD-PSH in 1981, 1992, 2002, and 2017.

	**Year**				
	**1981 (*N* = 85)**	**1992 (*N* = 196)**	**2002 (*N* = 214)**	**2017 (*N* = 294)**	***p***
**ADMISSION TO AN INPATIENT STRUCTURE**					
Immediate hospitalization	76.0%	34.2%	19.7%	15.6%	< 0.001
Delayed hospitalization	–	6.1%	1.6%	10.5%	< 0.001
**ADMISSION TO AN OUTPATIENT STRUCTURE**					
Community mental health service	–	43.9%	59.9%	59.2%	0.001
Post-emergency consultation	–	10.2%	15.9%	13.3%	0.235
**OTHER**					
Leave before orientation	–	0%	1.0%	0.3%	0.482
Hospitalization not accepted by the family	–	0%	1.9%	0%	0.014
No indication for follow-up	–	2.6%	0%	1.0%	0.046
Other	–	3%	0%	0%	< 0.001

*Data for the alternative to an immediate hospitalization were not systematically collected in 1981*.

## Discussion

### Study Strengths and Limitations

This study has a number of strengths, such as the assessment of emergency activities over more than three decades. But the study also has limitations. First, the naturalistic design of our study does not permit a standardized measure of clinical symptoms and diagnostic procedure that could result in assessment bias. Comparison of the discharge psychiatric diagnoses between the four time periods therefore requires caution. Second, as our study is monocentric selection bias exists. Therefore, findings should be interpreted in the context of the CAPD-PSH. For example, the development of specific care pathways for youths with severe developmental disorder in the CAPD-PSH explains that this group is underrepresented in our sample. Third, as a medical record review, the data were not originally collected for research purposes and therefore we may have under or overestimated certain patient characteristics based on data unavailability. Fourth, as no community help-seeking sample or no severity assessment was available here, we cannot properly tested whether the changes observed here are truly specific to emergency activity. Moreover, measures of duration of untreated illness, symptom severity and onset of illness (acute/insidious) would have given some insights to better understand the reasons for seeking consultation in emergency. Finally, the time intervals between each annual data can be seen as arbitrary. It cannot be excluded that significant changes have not been identified using a 10-year time scale. In particular, the 5-year period leading up 2017 has been of much interest given the evidence for marked increases in youth presenting for affective difficulties and suicidality during this time period in North America.

### Summary of the Main Results

We used naturalist clinician-reported data collected in 1981, 1992, 2002, and 2017 to examine the evolution of the types of youth mental health problems observed in an emergency unit in a child and adolescent psychiatric department over the last three decades.

In line with our first hypothesis, we found that since 1981 the number of patients presenting to the emergency service rose steadily, with an increase of 3.85-fold between 1981 and 2017. This is in line with the observations made in pediatric EDs in the US (5, 6, 14, 15), in Canada (4, 12), and in Spain (13). As mentioned in the introduction, in the context of the French national mental health system this finding supports the view that financial reasons are not a determining factor in the increase in the use of emergency services in children and adolescents. This is the first study using a period of observation extended over more than 30 years, compared with studies of 4 years (12, 14), 6 years (15), 9 years (6), and 10 years (4, 5). We found a constant increased use of emergency consultations over the study period, although not at the same pace, with the largest growth during the 1980s. To ensure that these numbers were not explained by demographic changes, we verified that the size of service catchment area of the CAPD-PSH remained stable over the study period using official demographic data (Table S1).

In line with our second hypothesis, we found that the proportion of consultations for a more frequently severe problem (e.g., delusion) did not increase to the same extent compared to common/more frequently benign forms of mental health problems (e.g., anxiety) over the study period. In fact, the number of consultations for delusion decreased between 1992 (8.7%, *n* = 17) and 2017 (4.4%, *n* = 13). Unfortunately, no specific measure of the severity of clinical cases (e.g., with triage or acuity assessments) was available here, while the use of the discharge diagnoses as a proxy measure for clinical severity has previously been used (4). We are uncertain whether this increased number of ED psychiatric diagnoses of mood and anxiety disorders reflects increased prevalence of these diseases, increased severity of these diseases, change in clinical practice with increased diagnosis in emergency consultation, heightened awareness of these diagnoses by the family the youth himself, or finally a decreased access to outpatient care for youths with mood and anxiety disorders. As our study did not assess currently available outpatient resources, clinical practices, or epidemiological data from non-help-seeking population the question remained unsolved. Although, in our study, preliminary evidence seem to show that the proportion of consultations for common/more frequently benign reasons have progressively increased during the last decades. This finding indirectly supports the assumption that the increased use of emergency structures in children and adolescents is partly explained by the shortages of mental health providers. Moreover, it is possible that the overutilization of emergency resources for the common/more frequently benign forms of mental health problems affect access to mental health emergency services for more severe form of mental illness.

We found in our sample that the consultations for anxiety and depressive mood have risen steadily over the three decades. This is in line with previous studies conducted in EDs (4, 12). This is also consistent with an increase in self-reported symptoms of depression during adolescence reported in general populations (22), and longitudinal data collected in both primary and secondary health care services (23). Interestingly, in our study such increase was not associated with a corresponding rise in the number of diagnosed depressive disorders. It may be due to changes in clinical practices, in particular the better recognition that mood symptoms can occur as part of emotional regulation difficulties in youths with externalizing problems (24). Unfortunately, this hypothesis cannot be properly tested as psychiatric comorbidities were not taken into consideration in this research. Moreover, anxiety disorders became the most frequent discharge psychiatric disorder in youths admitted in the emergency unit, rising from 5% in 1981 to 34% in 2017. In a context where consultations at the request of the family became the main source of referral, one might ask whether an increased attention to pediatric emotional disorders in scientific debate and in the general media could impact the way the parents expressed their concerns about their child, and ultimately increase the number of emergency visits for anxiety and depressive mood. However, it is worth remembering that the decrease in referral for suicidal behavior in emergency consultations over the last decades observed in our clinical sample does not tell us the evolution of this phenomenon in general population. In particular, given that the emergency consultation service studied is open only weekdays during daily hours and Saturday morning, the proportion of youth presenting for suicidal behavior is expected to be lower than in pediatric ED structure open overnight.

As illustrated by the Figures 1, 2, gender discrepancies in the main reasons for referral to the emergency consultation have been found: the visits for aggressive behaviors were more frequent for boys, while girls were more likely to consult for affective symptoms. Such findings are consistent with preexisting literature, (e.g., 5, 6, 15). Regarding our two principal hypotheses we did not find any significant gender effect, i.e., the growth in emergency consultations across time and the increase proportion of common/more frequently benign forms of mental health problems were observed both in boys and girls.

Between 1981 and 2017 the admission rate to an inpatient structure decreased both in absolute and relative terms, which is consistent with the political context of constant inpatient unit beds reduction for children and adolescents (9). For example the closure of child psychiatric inpatient facilities that took place in the 1970s led to a 70% decrease of the number of beds between 1986 and 2000 (9). The current admission rate to an inpatient structure in our study was lower than those reported in previous American studies [36% (4) and 52% (16)] but was comparable to those found in a Spanish sample [19% for (13)]. Interpretation of these findings should be cautious as all of these studies were conducted in 24 h/7 day pediatric EDs and not in a consultation service of a child and adolescent psychiatry department.

Our finding supports a shift to a more comprehensive use of alternatives to direct hospitalization. In 2017, the discharge to a community mental health service was the most frequent outcome after the emergency visit. It should be noted that the delay for an appointment in a community mental health service varies greatly across Paris areas, and there are virtually no outpatient structures in France that can provide same-day evaluations. It appears that one out of 10 patients presenting to the emergency consultations was admitted to a pediatric inpatient unit until a bed in a child and adolescent psychiatric ward became available. The extent of this practice (called “delay hospitalization”) is not without consequences on the quality of the mental health care provided (7). The development of specific training for the medical and paramedical pediatric teams who receive these patients represents a possible area of improvement (2, 25).

### Research and Clinical Implications

We have confirmed the growing use of mental health emergency consultations and the changes in the types of mental health problems of children and adolescents over the last three decades. Indirect evidence support the assumption that this change is caused by growing patient and family demands in a context of shortage of mental health providers rather than demographic, economic or epidemiological changes. Further research should (i) use specific measures of clinical severity and comorbid psychopathology and (ii) contrast the finding from a help-seeking sample with a community-based sample. This would help to more precisely understand the socio-demographic and clinical factors that contribute to the evolution observed. These findings may, in turn, aid the development of intervention in the model of programs being developed and piloted across the country to provide quick evaluations in an outpatient setting (26).

## Conclusions

In conclusion, using a comparison of data collected at four periods over the last three decades, we found that demographic and clinical changes exist in the types of youth mental health problems presenting to emergency in a context of increasing demand. In particular increasing proportions of emergency consultations are motivated by anxiety and depressive mood and are directly requested by the family. This highlights the need to develop scientific research and community healthcare networks to better understand and address the issue of emergency-unit overcrowding.

## Author Contributions

XB, VM, and DC: substantial contributions to the conception and design of the work; XB, VM, AO, SV, and NE: substantial contributions to the acquisition, analysis, or interpretation of data; XB, VG, and DC: drafting the work or revising it critically for important intellectual content; XB, VM, AO, SV, NE, VG, J-MG, and DC: final approval of the version to be published and agreement to be accountable for all aspects of the work in ensuring that questions related to the accuracy or integrity of any part of the work are appropriately investigated and resolved.

### Conflict of Interest Statement

The authors declare that the research was conducted in the absence of any commercial or financial relationships that could be construed as a potential conflict of interest.
