# Supplementary material for: Changes in the Use of Emergency Care for the Youth With Mental Health Problems Over Decades: A Repeated Cross Sectional Study
Source: Front Psychiatry. 2019 Feb 6;10:26. doi: 10.3389/fpsyt.2019.00026 (PMC6372506; doi:10.3389/fpsyt.2019.00026)
Supplement: Supplementary file 1 [file Table_1.docx]

|  |  | Year | | | |
| --- | --- | --- | --- | --- | --- |
|  |  | 1981 | 1992 | 2002 | 2017 |
|  | Service Catchment Area^1^ | 269,970 | 267,970 | 270,507 | 278,241 |

**Table S1. Evolution of the service catchment area, inpatient annual admission rate, and annual admission of patients under 18 to the adult ED during the period of the study**

^1^ Based on the INSEE demographic evaluation of the population
